# ﻿ *Primulasurculosa* (Primulaceae), a new species from Yunnan, China

**DOI:** 10.3897/phytokeys.212.91133

**Published:** 2022-10-28

**Authors:** Yuan Xu, De-Ming He, Lin-Zhong Yang, Gang Hao

**Affiliations:** 1 Key Laboratory of Plant Resources Conservation and Sustainable Utilization, South China Botanical Garden, Chinese Academy of Sciences, Guangzhou 510650, China South China Botanical Garden, Chinese Academy of Sciences Guangzhou China; 2 Center of Conservation Biology, Core Botanical Gardens, Chinese Academy of Sciences, Guangzhou 510650, China Center of Conservation Biology, Core Botanical Gardens, Chinese Academy of Sciences Guangzhou China; 3 Administration of Wenshan National Natural Reserve, Wenshan Zhuang and Miao Autonomous Prefecture 66300, China Administration of Wenshan National Natural Reserve Wenshan Zhuang and Miao Autonomous Prefecture China; 4 College of Life Sciences, South China Agricultural University, Guangzhou 510642, Guangdong, China South China Agricultural University Guangzhou China

**Keywords:** morphological characteristics, new species, *Primulataliensis*, taxonomy, Yunnan

## Abstract

A new species, *Primulasurculosa*, is described and illustrated. In gross morphology, it is clearly allied to section Petiolares and is most similar to *P.taliensis* from the group *Taliensis*, but is distinctive in its indumentum in the throat of the corolla tube, and the markedly stoloniferous habit.

## ﻿Introduction

The genus *Primula* L. (Primulaceae) is one of the rapid speciation groups in angiosperms, which comprises about 500 species that are almost exclusively confined to temperate and arctic zones of the Northern Hemisphere. Its modern center of diversity falls in the Hengduan-Himalayan region, harboring more than 300 species (Hu 1990; Hu and Kelso 1996). Its ancestral or original center, however, is speculated to be in the montane region of SW China (southern Yunnan, Guizhou and Guangxi) and adjacent northern Vietnam, Myanmar and Thailand, since many presumably primitive taxa of *Primula*, *Lysimachia* and *Androsace* (Primulaceae) occur there (Hu 1994; Hao et al. 2004).

Primulasect.Petiolares Pax has approximately 60 species worldwide, and is abundantly distributed in the Hengduan-Himalaya Mts., with only a few members extending into central China, N Myanmar, N Vietnam and Kashmir (Hu 1990; Hu and Kelso 1996). One of the most important diagnostic characters of this section is its globose capsule with a persistent calyx that does not open by valves but by crumbling at the membrane apex (Smith and Fletcher 1944; Hu 1990). This section was further divided into seven groups based on the presence or absence of the basal bud scales and farina, the shape of the leaf margin, and the type of hair (Smith and Fletcher 1944).

In the spring of 2020, while the authors were investigating flora of *Primula* in southeastern Yunnan, a population of *Primula* was discovered in Wenshan city. In the flowering time, particularly from the appearances of leaves and flowers, it looked a bit like *Primulataliensis*, which occurs in western Yunnan and northern Myanmar. In the following fruiting time, the plants notably developed several leafy stolons. Detailed examination proved that it eventually represents an unreported taxon of P.sect.Petiolares, and is described below.

## ﻿Materials and methods

Firstly, we examined the relevant taxonomic literature (e.g., Smith and Fletcher 1944; Hu 1990; Hu and Kelso 1996) to infer the similar species for the new species and the main diagnostic characters (habit, the indumentum of leaves and corolla, the shape of leaves, calyx lobe and corolla lobe, and the length of petiole) which should be compared. Then, the observations and measurements of morphological characters of the new taxon were conducted in the field and at the herbarium. Indumentum and other tiny morphological features were observed under a stereomicroscope. Flowers were dissected and photographed. Morphological comparison with similar species was performed based on living plants (for *P.taliensis* collected from Jingdong and Dali of Yunnan Province), specimens from IBSC, KUN and PE, and the images of specimens from the JSTOR Global Plants (http://plants.jstor.org/). The conservation status of the new species was assessed following the guidelines for using the IUCN Red List categories and criteria (IUCN Standards and Petitions Subcommittee 2022).

## ﻿Taxonomic treatment

### ﻿ Primula surculosa

Taxon classificationPlantaeEricalesPrimulaceae

Y.Xu & G.Hao sp. nov.

B74134F4-35AC-5ECA-BCB1-E4FDAED45949

urn:lsid:ipni.org:names:

Figs 1 , 2

#### Type.

China, Yunnan: Wenshan City, Bozhu Town, Bozhu Mt. 23°22'N, 104°12'E, alt. 2910 m, 27 Feb. 2022 (fl.), Deming He Xu211011 (holotype: IBSC!).

#### Diagnosis.

*Primulasurculosa* is morphologically most similar to *P.taliensis*, but is distinctive in its indumentum in the throat of the corolla tube, and the markedly stoloniferous habit.

#### Description.

A perennial herb, efarinose, stoloniferous, lacking basal bud scales at anthesis. ***Leaves*** dimorphic, forming a rosette of 9.0–2.0 cm in diameter, with short appressed pubescent on both surfaces. ***Outer leaves*** spatulate to obovate-spatulate, 2.5–5.0 × 2.5–3.5 cm, tapering to base forming a broadly winged petiole, margin crenate to dentate, apex rounded. ***Inner leaves*** long petiolate; blade broadly ovate to suborbicular in outline, 4.0–7.0 cm in diameter, base rounded or cordate, margin coarsely dentate; petiole 3.0–6.0 cm long, up to 12 cm at fruiting time. ***Stolons*** arising from the basal of the leaf rosette after anthesis, terminated in a leaf rosette, with alternate and reduced ovate leaves (5–18 × 8–20 mm) on lower part, growing upwards to 5–8 cm, afterwards elongating up to 20–25 cm long, procumbent along the surface of the ground and rooting at the nodes. ***Scapes*** 1.5–3.0 cm, reaching 6.0 cm at fruiting, copiously pilose; umbel solitary, 2–8 flowered; bracts lanceolate, 3–6 mm. ***Pedicel*** 2–3 cm. ***Flowers*** heterostylous. ***Calyx*** campanulate, 6–8 mm, parted to 1/3; lobes ovate to broadly lanceolate, margin 3-toothed at apex and occasionally entire in fruiting. ***Corolla*** purplish rose to purple-blue; tube 8–12 mm, with a tuft of white hairs projecting the yellowish green annulus in throat; limb 1.2–1.6 cm wide; lobes broadly obovate, 3-toothed. ***Pin flowers***: stamens 5–6 mm above base of corolla tube; style nearly as long as tube. ***Thrum flowers*** with positions reciprocal. ***Capsule*** subglobose, included in calyx, disintegrating at maturity.

#### Distribution and habitat.

The new species is presently known only from the type locality in Yunnan, Wenshan City, and is clustered in small groups in deep moss under secondary evergreen broad-leaved forests.

#### Phenology.

Flowering from February to April, fruiting from April to June.

#### Etymology.

The specific epithet “surculosa” refers to the remarkable root-suckers (stolons), with long slender internodes and reduced leaves arising after anthesis.

#### Conservation status.

Based on our field investigations in Wenshan City and adjacent regions (e.g., Pingbian, Maguan, Malipo and Mengzi) during the last three years, only one population with ca. 800 individuals of the new species has been found in an area of 10 km^2^. Moreover, according to the result of our investigation in the villages near the type locality, the local folks often collect this new species as a medicinal plant. Therefore, the conservation status of the new species is assessed as vulnerable (VU D1+2) according to the guidelines for using the IUCN Red List categories and criteria (IUCN Standards and Petitions Subcommittee 2022).

#### Additional specimens examined (paratypes).

The same locality as holotype, 9 May 2021, Deming He Xu210577 (IBSC!); 26 April 2022, Deming He Xu211017 (IBSC!); 2 July 2022, Deming He Xu221030 (IBSC!).

#### Relationship with related species.

Group *Taliensis* is a small group of two species (*P.taliensis* and *P.comata*) in sect. Petiolares, characterized by plants without basal bud scales at anthesis, scape equaling or exceeding the leaves at flowering time, plant glandular hairy, and efarinose (Smith and Fletcher 1944). This group is mainly distributed in western Yunnan and adjacent northern Myanmar (Smith and Fletcher 1944). The present new species is assigned to this group, being distinctive in the markedly stoloniferous habit, and some other morphological features, which are summarized in Table 1.

**Table 1.** Main morphological differences between *Primulasurculosa* and two similar species (Hu 1990; Hu and Kelso 1996).

Features	*P.surculosa*	*P.taliensis*	*P.comata*
Stolon	present	absent	absent
Leaves			
Indumentum	short appressed pubescent	short appressed pubescent	long fulvous hairs
Inner blade shape	ovate to suborbicular	ovate-rounded to reniform	elliptic
Petiole of inner leaves at fruiting	2–3 times as long as blade	1–2 times as long as blade	slightly longer than blade
Calyx lobe apex	3-teethed, occasionally entire in fruiting	subacuminate to acute, occasionally denticulate	obtuse to rounded
Corolla lobe apex	3-teethed	3-teethed	entire
Throat of the corolla tube	pilose	glabrous	pilose

The stolon, an unusual mechanism of vegetative propagation, is an outstanding feature of the new species. However, this feature appears to have multiple origins in this genus, since it also occasionally occurs in some species of other sections which are presumably not intimately connected, e.g., *P.heucherifolia* (sect. Cortusoides Balf. f.), *P.ranunculoides* (sect. Ranunculoides Chen et C.M.Hu), *P.caldaria* (sect. Aleuritia Duby), and *P.pseudodenticulata* (sect. Denticulata Watt) (Hu 1990; Hu and Kelso 1996; Shao et al. 2012). So the stolon may have no phylogenetic significance in the genus *Primula*.

**Figure 1.**
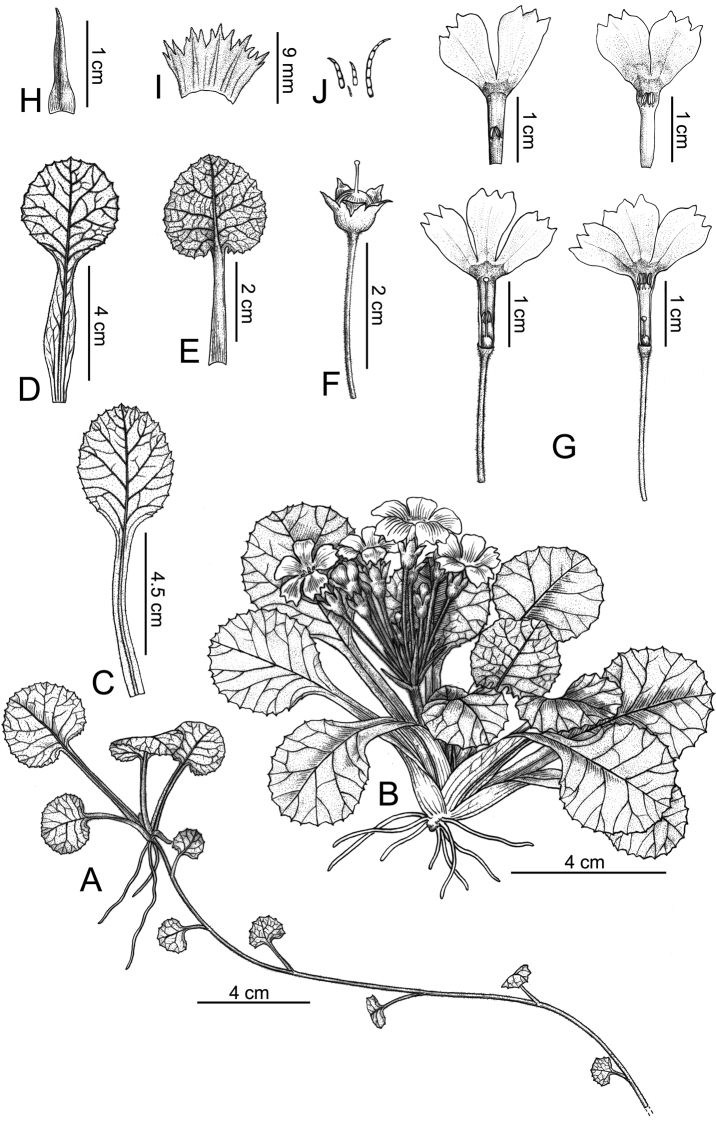
*Primulasurculosa* sp. nov. **A** stolon **B** habit **C** leaf on abaxial surface (fruiting time) **D** outer leaf on abaxial surface (anthesis) **E** inner leaf on adaxial surface (anthesis) **F** capsule with persistent calyx **G** long and short-styled flowers **H** bract **I** calyx (dissected) **J** multicellular hairs. Drawn by Yun-Xiao Liu.

**Figure 2.**
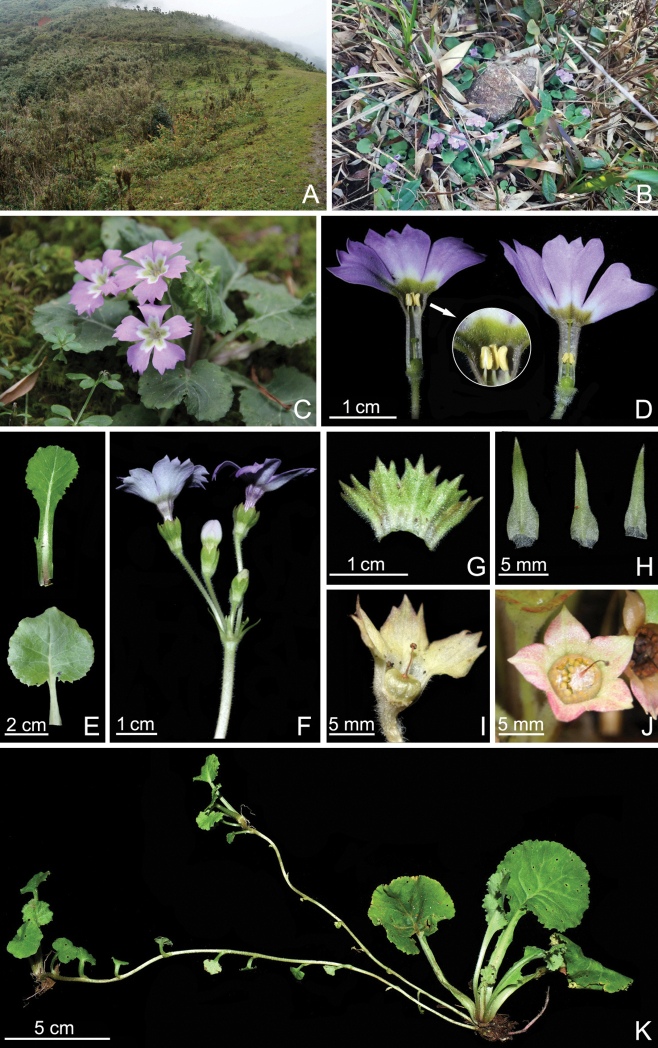
Living plant of *Primulasurculosa* sp. nov. **A, B** habitat **C** habit (blooming) **D** long and short-styled flowers, also showing pilose corolla tube (the circular image) **E** outer and inner leaves on adaxial surfaces, showing indumentum, venation, and margin shapes **F** inflorescence **G** calyx (dissected) **H** bracts **I** capsule with persistent calyx **J** capsule (crumbling) **K** habit (stoloniferous after anthesis) Photographed by De-Ming He.

## Supplementary Material

XML Treatment for Primula surculosa
